# STN1 Shields CTC1 From TRIM32‐Mediated Ubiquitination to Prevent Cellular Aging

**DOI:** 10.1111/acel.70214

**Published:** 2025-09-09

**Authors:** Yina Lan, Xiaole Liang, Guotao Kuang, Tengfei Ma, Fangyingnan Zhang, Zaoli Huang, Huan Wang, Zhenhua Luo, Xuyang Feng

**Affiliations:** ^1^ Department of Pediatrics, The First Affiliated Hospital Sun Yat‐Sen University Guangzhou Guangdong China; ^2^ Institute of Precision Medicine, The First Affiliated Hospital Sun Yat‐Sen University Guangzhou Guangdong China

**Keywords:** cell senescence, CST, CTC1, STN1, TRIM32, ubiquitin

## Abstract

The CST (CTC1‐STN1‐TEN1) complex, a single‐stranded DNA (ssDNA) binding complex, is essential for telomere maintenance and genome stability. Depletion of either CTC1 or STN1 results in cellular senescence, while mutations in these components are associated with severe hereditary disorders. In this study, we demonstrate that the direct STN1‐CTC1 interaction stabilizes CTC1 by preventing its degradation via TRIM32 mediated ubiquitination. Functional assays indicate that TRIM32 and the CTC1/STN1 complex exert opposing effects on cellular proliferation. Additionally, transcriptomic analysis of large‐scale RNA sequencing data from the Genotype‐Tissue Expression (GTEx) reveals inverse expression patterns of TRIM32 and CTC1/STN1 during somatic cell aging. Structural modeling using AlphaFold3 predicts that the TRIM32‐CTC1 interaction occurs at the OB‐G domain of CTC1, with the binding interface positioned near the STN1‐interacting region, termed the “cleft” motif. Mechanistically, STN1 likely associates with the OB‐G domain of CTC1, competing with TRIM32 for binding sites and thereby interfering with TRIM32‐mediated ubiquitination of CTC1. Collectively, our findings identify STN1 as a critical regulator of CST complex integrity and cellular aging by safeguarding CTC1 from TRIM32‐driven ubiquitin‐proteasome degradation.

## Graphical abstract

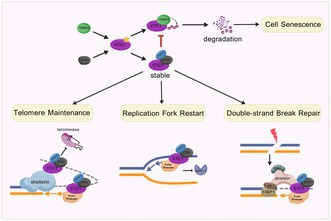

## 1 Introduction

Genomic instability and telomere attrition are two of the most common hallmarks of aging (López‐Otín et al. 2023). Throughout life, the stability of the human genome is continuously challenged by the accumulation of DNA damage from exogenous and endogenous threats, such as chemical agents and DNA replication errors (Magdalou et al. 2014; Ragu et al. 2020; Zeman and Cimprich 2014). The accumulated DNA damage triggers cellular senescence and ultimately leads to cell death (Coluzzi et al. 2019; Gorbunova et al. 2021).

Telomeres are nucleoprotein complexes located at the ends of eukaryotic chromosomes. Mammalian telomere DNA consists of kilobases of double‐stranded 5′‐TTAGGG‐3′ (G‐strand) 3′‐AATCCC‐5′ (C‐strand) repeats and terminates with a 12–400 nt 3′ G‐rich overhang (termed the G‐overhang) (Makarov et al. 1997; Moyzis et al. 1988). Stem cells and most cancer cells maintain their telomere length through a telomerase‐initiated telomere elongation machinery (Allen and Baird 2009; Blasco 2005). However, in normal human somatic cells, telomeres shorten with each round of cell division due to the end replication problem (Harley et al. 1990). The telomere length of somatic cells has been found to correlate with human age (Blasco 2005).

Besides acting as a buffer for genetic information lost during cell division, telomeres play critical roles in chromosome protection and genome stability (Frias et al. 2012). The telomere G‐overhang serves as an important structure not only for telomerase binding in telomerase‐positive cells but also for preventing linear chromosomes from illegitimate degradation or end‐to‐end fusion by inserting into the duplex region to form a telomere loop (T‐loop) (Ruis and Boulton 2021). Therefore, telomere duplex shortening or G‐overhang destruction limits proliferative potential and contributes to cellular senescence (Lim and Cech 2021).

CST (CTC1‐STN1‐TEN1) is a single‐stranded DNA binding complex with a preference for G‐rich sequences (Hom and Wuttke 2017), functioning in both telomere maintenance and genome stability (Chastain et al. 2016; Miyake et al. 2009). At the telomeres, CST engages DNA polymerase at chromosome ends to fill in the C‐strands and prevents G‐quadruplex accumulation to assist in duplex replication (Takai et al. 2024; Zhang et al. 2019). Elsewhere in the genome, it helps recover stalled replication forks and facilitates DNA damage repair (Mirman et al. 2022; Stewart et al. 2012). Moreover, in telomerase‐positive cells, CST mediates telomerase termination after G‐strand elongation to maintain appropriate G‐overhang lengths (Feng et al. 2018; Gu et al. 2018). Given the essential functions of CST, depletion of the complex in cells results in early senescence, and mutations of its components in individuals cause serious genetic disorders, such as dyskeratosis congenita and Coats Plus syndrome (Gu et al. 2018; Revy et al. 2023; Y. Wang and Chai 2018).

With a 1:1:1 subunit stoichiometry in vivo, the integrity of the complex is critical for CST (Lim et al. 2020). Knockdown or knockout of TEN1 reduces DNA binding affinity and causes failure in C‐strand fill‐in (Bryan et al. 2013; Feng et al. 2018). Depletion of CTC1 or STN1, or disruption of the STN1‐CTC1 interaction, results in loss of DNA binding ability, ultimately leading to the incapacity to perform its functions and subsequent cell senescence (Chen et al. 2013; Feng et al. 2017; Gu et al. 2018; Huang et al. 2012). Previous studies have shown that reduction of one component may decrease the levels of the others (Feng et al. 2017; Gu et al. 2012). However, it remains unclear how the three subunits stabilize each other and sustain their complex formation.

Post‐translational modifications (PTMs) of CST subunits play crucial roles in regulating their functions in yeast cells. SUMOylation of Cdc13, the homolog of human CTC1, facilitates its interaction with Stn1 and acts as a negative regulator of telomerase activity in budding yeast (Hang et al. 2011). Phosphorylation of Cdc13 by MEC1p and TEL1p inhibits its interaction with telomerase (Tseng et al. 2006), whereas Cdk1‐mediated phosphorylation at T308 enhances its telomerase binding and promotes telomere elongation (Tseng et al. 2009). Additionally, phosphorylation of Stn1 at T223/S250 in budding yeast strengthens its interaction with Cdc13, while phosphorylation at S74 in fission yeast reduces its telomere‐binding affinity (Liu, Gopalakrishnan, et al. 2014). These findings suggest the potential influence of PTMs on the regulation of human CST. Notably, phosphorylation of STN1 has recently been implicated in the protection of stalled replication forks (Jaiswal et al. 2023). Here, we demonstrate that ubiquitination of CTC1 leads to its degradation via the proteasome pathway, while STN1 counteracts CTC1 ubiquitination and stabilizes the CST complex by competitively inhibiting the binding of ubiquitin E3 ligase to CTC1. This study identifies TRIM32 as a novel regulator of CST homeostasis and emphasizes the critical role of CST integrity in preventing cellular senescence.

## 2 Methods

### 2.1 Cell Culture and Generation of Stable Cell Lines

HEK293T cells and BJ fibroblast cells were grown in DMEM medium, supplemented with 10% FBS and antibiotics. Sf9 cells were cultured in Grace' Insect Medium (Gibco) containing 10% inactivated FBS. To introduce wild‐type or mutant Flag‐CTC1, retrovirus was co‐transfecting HEK293T cells with the pMIEG3 vector encoding wild‐type or mutant Flag‐CTC1, gag‐pol, and env. Viral supernatant was used to infect HEK293T cells, and the cells were then selected by flow cytometry for GFP expression. To introduce TRIM32, lentivirus was co‐transfecting HEK293T cells with pLenti‐EF1a‐C‐SFB vectors encoding TRIM32, pMD2.G, and psPAX2. Viral supernatant was used to infect HEK293T cells, and the cells were then selected by puromycin.

### 2.2 RNA Interference

Small interfering RNA (siRNA) oligonucleotides were transfected into cells using RNAiMax reagent (ThermoFisher) for 48–72 h. The sequences are: siControl: 5′‐UUCUCCGAACGUGUCACGU‐3′; siSTN1‐1: 5′‐GAUCCUGUGUUUCUAGCCU‐3′; and siSTN1‐2: 5′‐GCUUAACCUCACAACUUAA‐3′.

### 2.3 Co‐Immunoprecipitation (Co‐IP) and Western Blotting (WB)

Co‐IPs were performed as previously described (H. Wang et al. 2023). Briefly, HEK293T cells expressing the indicated proteins were lysed in NP‐40 lysis buffer (20 mM Tris pH 8.0, 100 mM NaCl, 1 mM MgCl_2_, and 0.1% Igepal). For immunoprecipitation, each sample was incubated with Flag M2 beads (Sigma) or HA beads (Sigma) at 4°C for 2 h, washed with NP‐40 lysis buffer, and boiled in 2× SDS loading buffer. Samples were separated by SDS‐PAGE and transferred to nitrocellulose membranes. The membranes were blocked with 5% milk in TBS‐Tween (0.1% Tween‐20) and incubated with antibodies to Flag (Cell Signaling Technology), HA (Affinity Biosciences), GST (Proteintech), CTC1 (homemade), STN1 (Abcam), ubiquitin (Cell Signaling Technology), GFP (Abcam), p53 (Santa Cruz), Lamin B1 (Proteintech), Ki‐67 (Abmart), α‐actinin (Proteintech), and GAPDH (Proteintech).

### 2.4 Protein Purification

BL21 (DE3) *E. coli* cells were transfected with pGEX5 vector encoding GST‐tagged TRIM32 or pET‐28a vector encoding His‐tagged TRIM32, cultured overnight at 16°C with 0.5 mM isopropyl β‐D‐1‐thiogalactopyranoside, and lysed with NP‐40 lysis buffer (0.1% Igepal, 20 mM Tris–HCl pH 8.0, 100 mM NaCl, 1 mM MgCl_2_). The supernatant was incubated with Glutathione‐sepharose beads (Cytiva) or Nickel beads (Qiagen) for 1 h. The beads were then washed three times and eluted with elution buffer containing 10 mM reduced glutathione or 200 mM imidazole (Sigma). The purified protein concentration was quantified by Coomassie blue staining.

For baculovirus expression, Sf9 cells in suspension were infected with baculovirus co‐expressing Flag‐CTC1 and His‐STN1. The infected cells were cultivated in a 500 mL shaker flask (1 × 10^6^ cells/mL) at 130 rpm for 72 h at 27°C. Subsequently, cells were harvested and resuspended in NP‐40 lysis buffer with protease inhibitors. After incubation at 4°C for 10 min, the lysate was sonicated using a non‐contact ultrasonic crusher and centrifuged. The Flag M2 beads, washed and pre‐equilibrated with TBS buffer (50 mM Tris–HCl, pH 7.4, 150 mM NaCl), were added to the supernatant and incubated for 1 h at 4°C. The beads were then washed three times with TBS buffer and eluted using Flag elution buffer (Tris–HCl, pH 7.5, 300 mM NaCl, 10% glycerol, 1 mM DTT, 0.15 mg/mL 3xFLAG peptide (Beyotime)) at 4°C for 30 min. The purified protein concentration was quantified by silver staining.

### 2.5 In Vitro Pull‐Down Assays

*E. coli* cells expressing GST‐TRIM32 were lysed with NP‐40 lysis buffer and incubated with Glutathione‐sepharose beads for 2 h at 4°C. The beads were washed, added to the lysis from Sf9 cells expressing Flag‐CTC1, and incubated for 4 h at 4°C. After washing, the precipitates were collected and analyzed by WB.

### 2.6 In Vivo Ubiquitination Assays

HEK293T cells were co‐transfected with His‐Ub WT or K48 and the indicated constructs. After 40 h, the cells were treated with 10 μM carbobenzoxy‐Leu‐Leu‐leucinal (MG132) and cultured for another 8 h before harvesting. Cells were lysed in buffer A (6 M guanidine‐HCl, 0.1 M Na_2_HPO_4_/NaH_2_PO_4_ and 10 mM imidazole (pH 8.0)) and sonicated for six cycles (1 s on/1 s off). Following centrifugation at 15,000 *g* for 10 min, the supernatants were collected and incubated with Ni–NTA beads for 3 h at RT. The beads were washed sequentially with buffer A, buffer A/Ti (buffer A and buffer Ti mixed in a 1:3 ratio), and twice with buffer Ti (25 mM Tris–HCl (pH 6.8) and 20 mM imidazole). Then, the precipitated products were separated by 6% SDS‐PAGE gels and analyzed with Western blotting.

### 2.7 In Vitro Ubiquitination Assays

Flag‐CTC1 co‐expressed with His‐STN1 and purified from Sf9 cells was incubated with *E. coli* cells purified His‐TRIM32, along with E1, E2 (UbcH5a), and K48‐linked ubiquitin (UB‐biotech) in the reaction buffer for 3 h at 37°C. The samples were then boiled in 2× SDS loading, separated by 6% SDS‐PAGE gels, and analyzed with WB.

### 2.8 Mass Spectrometry

Flag‐IP products from HEK293T cells expressing Flag‐CTC1 were used for identifying CTC1 interacting proteins. For ubiquitination sites identification, Flag‐IP was performed with Flag‐CTC1 expressing HEK293T cells co‐transfected with/without TRIM32, treated with MG132 prior to harvesting. The products were separated by SDS‐PAGE and stained with Coomassie blue. CTC1 bands were sectioned for subsequent trypsin digestion, peptide extraction, and mass spectrometry sequencing. The IP products or sectioned gels were sent to Wininnovate Biotechnology Co. Ltd. in Shenzhen for further processing. The liquid chromatography–tandem mass spectrometry was performed with a Triple TOF 6600 tandem mass spectrometer (Sciex, Concord, Ontario, Canada).

### 2.9 Bimolecular Fluorescence Complementation (BiFC) Assays

Sequences encoding CTC1 and TRIM32 were cloned into either the C‐ or N‐terminal fragment of YFP (FG‐EH‐YFPc‐DEST2‐PPW or FG‐EH‐YFPn‐DEST2‐PBW) vectors via Gateway recombination. YFP signals were detected and quantified 48 h post‐transfection using flow cytometry.

### 2.10 Cycloheximide (CHX) Chase Assays

HEK293T cells stable expressing wild‐type or mutant Flag‐CTC1 were transfected with the indicated plasmids and treated with CHX (100 μg/mL) for the specified time 48 h post‐transfection before harvesting. Protein levels of CTC1 were detected by WB with anti‐Flag antibodies and quantified with e‐BLOT Touch Imager analysis software.

### 2.11 Senescence‐Associated β‐Galactosidase (SA‐β‐Gal) Staining

SA‐β‐gal activity in cells was detected using a staining kit (Beyotime) according to the manufacturer's instructions. Briefly, cells were seeded in 6‐well plates. After 24 h, cells were fixed and incubated with β‐galactosidase staining solution overnight at 37°C. Images were taken with a Zeiss microscope.

### 2.12 Correlation Analysis of Gene Expression With Age

Gene expression data were from the large RNA‐seq dataset of the Genotype‐Tissue Expression (GTEx) project (The Genotype‐Tissue Expression (GTEx) Project 2013) and downloaded from voyAGEr website (Schneider et al. 2024). The graphical results were generated using GraphPad Prism.

### 2.13 Real‐Time Cell Proliferation Analysis

BJ cells were seeded on 16‐well e‐plates at a density of 1 × 10^4^ cells/well, placed in the iCELLigence real‐time cell analyzer (RTCA) DP Instrument (ACEA Biosciences), then incubated in a CO_2_ incubator at 37°C for 7 days. The cell index was registered every 30 min, calculated by the RTCA Software Pro, and depicted as a function of time.

### 2.14 AlphaFold3 (AF3) Structural Analysis

Protein sequences for human CTC1, TRIM32, and TRIM32‐NHL were analyzed using AF3, resulting in the generation and ranking of five models of the CTC1‐TRIM32 complex. The model with the highest score was visualized using MolStar on the RCSB PDB web site.

### 2.15 Schematic Illustrations

Schematic illustrations and working models were created using the Generic Diagramming Platform (Jiang et al. 2025).

### 2.16 Statistical Methods

Data from a minimum of three independent experiments were subjected to analysis using Student's *t*‐test and two‐way ANOVA test. Significant differences were defined as *p < 0.05, **p < 0.01.

## 3 Results

### 3.1 STN1 Maintains CTC1 Level by Suppressing Its Ubiquitin‐Proteasome Degradation

**FIGURE 1:**
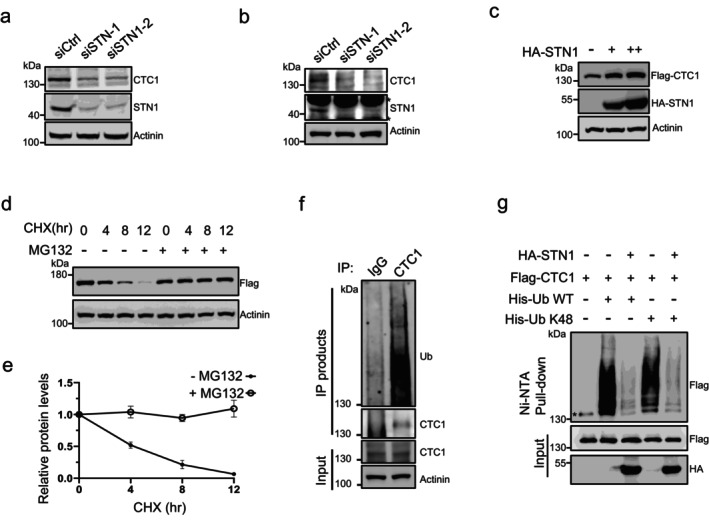
STN1 maintains CTC1 levels by suppressing its ubiquitin‐proteasome degradation. (a, b) Immunoblot showing CTC1 protein levels upon disruption of STN1 by siRNAs in HCT116 (a) or BJ fibroblast cells (b) *: Cross‐reacting bands. (c) Immunoblot showing Flag‐CTC1 levels in HEK293T cells transfected with increasing amount of haemagglutinin (HA)‐tagged STN1. (d) Represent immunoblot showing Flag‐CTC1 and control protein levels over a 12‐h period with or without MG132 in the presence of CHX to inhibit protein synthesis. (e) Quantification of relative Flag‐CTC1 levels from (d). *N* = 3 independent experiments. Error bars represent mean ± S.E.M. (f) Immunoblots showing ubiquitin levels in immunoprecipitates obtained using CTC1‐specific antibodies or IgG control from HEK293T cells. (g) In vivo ubiquitination assays showing STN1's effect on wild‐type (WT) and K48‐linked (K48) CTC1 ubiquitination levels. Immunoblot of nickel‐nitrilotriacetic acid (Ni‐NTA) pull‐downs and whole‐cell inputs from HEK293T cells co‐transfected with Flag‐CTC1, His‐ubiquitin (His‐Ub) (WT or K48) and HA‐STN1. *: Non‐specific binding to Ni‐NTA resin. Western blots were performed with antibodies against CTC1, STN1, ubiquitin, Flag and HA.

Upon the disruption of STN1 expression using two independent small interfering RNAs (siRNAs), we observed a significant reduction in CTC1 levels in both telomerase‐positive HCT116 colon cancer cells and telomerase‐negative BJ fibroblasts (Figure 1a,b). To further investigate the protein levels and mitigate the interference from background signals of endogenous CTC1 antibodies, we established a stable HEK293T cell line expressing Flag‐tagged CTC1. By transfecting varying amounts of HA‐tagged STN1 expression constructs into these cells, we noted a progressive increase in Flag‐CTC1 correlating with the levels of STN1 overexpression (Figure 1c). These findings indicate that STN1 plays a crucial role in maintaining CTC1 protein levels in vivo.

Since that protein levels are regulated by both synthesis and degradation, we examined the two processes in relation to CTC1 separately. Initially, we assessed the mRNA levels of CTC1 through real‐time PCR, revealing that neither STN1 depletion nor overexpression significantly impacted CTC1 synthesis (Figure S1a,b), effectively ruling out STN1's role in CTC1 production. Subsequently, we investigated CTC1 degradation by administering cycloheximide (CHX) to inhibit protein synthesis. In our degradation assays, we observed a gradual decline in Flag‐CTC1 levels over a 12‐h period with a half‐life of about 3.5 h. Notably, the proteasome inhibitor MG132 effectively mitigated this decrease, suggesting that CTC1 is predominantly degraded via the proteasome (Figures 1d,e and S1c). Given that the ubiquitin‐proteasome pathway is the classical mechanism for protein degradation, we first performed immunoprecipitation of endogenous CTC1 from HEK293T cells using CTC1‐specific antibodies. As shown in Figure 1f, ubiquitin was detected in the immunoprecipitated CTC1 complex using ubiquitin‐specific antibodies, indicating that CTC1 is ubiquitinated under physiological conditions. Then, we proceeded to evaluate the ubiquitylation of CTC1 in vivo ubiquitylation assays. Co‐expressing Flag‐CTC1 with a His‐tagged ubiquitin (His‐Ub) construct in HEK293T cells and enriching ubiquitinated proteins using Ni‐NTA resin revealed that Flag‐CTC1 was specifically enriched on the NTA, indicating that CTC1 is subject to ubiquitin modification (Figure 1g). Furthermore, we observed that STN1 inhibits CTC1 ubiquitylation, as the enrichment of Flag‐CTC1 on His‐Ub NTA markedly decreased following the introduction of HA‐STN1. Since ubiquitin‐proteasome degradation is typically mediated by Lysine 48 (K48)‐linked ubiquitin, we repeated the ubiquitylation assays utilizing a K48‐only ubiquitin construct. A similar enrichment pattern of Flag‐CTC1 was observed, along with a consistent effect of STN1 (Figure 1g). Collectively, these data suggest that STN1 stabilizes CTC1 by inhibiting its proteasomal degradation through K48‐linked ubiquitylation.

### 3.2 STN1 Stabilizes CTC1 Through Their Physical Interaction

**FIGURE 2:**
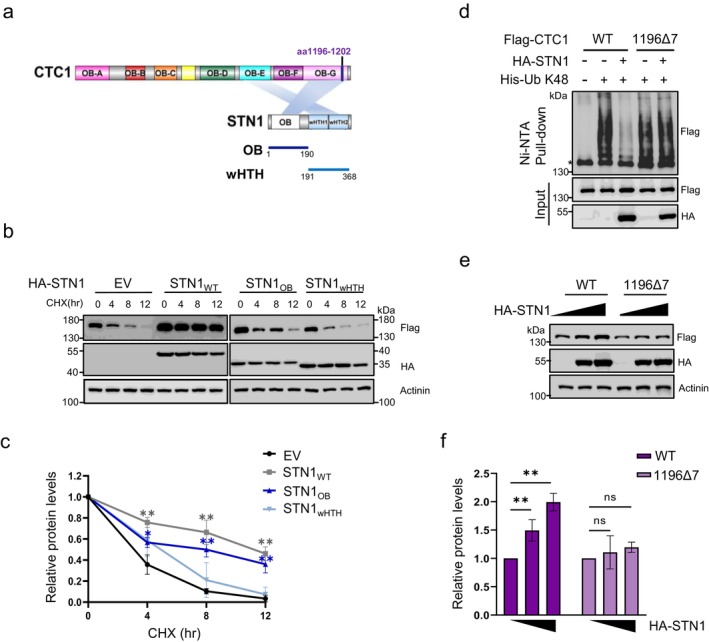
STN1 stabilizes CTC1 through their physical interaction. (a) Schematic representation of the interacting regions of CTC1‐STN1 and the design of mutants. (b) Protein degradation analysis of Flag‐CTC1 in HEK293T cells transfected with the indicated HA‐STN1 mutants over a 12‐h period. (c) Quantification of relative Flag‐CTC1 levels from (b). (d) In vivo ubiquitination assays showing STN1's effects on the K48 ubiquitin level of WT and 1196Δ7 mutated Flag‐CTC1. Immunoblot of Ni‐NTA pull‐downs and whole‐cell inputs from HEK293T cells co‐transfected with WT or 1196Δ7 Flag‐CTC1, His‐Ub and HA‐STN1. *: Non‐specific binding to Ni‐NTA resin. (e) Immunoblot showing the protein level of WT or 1196Δ7 Flag‐CTC1 in HEK293T cells transfected with increasing amounts of HA‐STN1. (f) Quantification of relative Flag‐CTC1 levels from (e). *N* = 3 independent experiments. Error bars represent mean ± S.E.M. Western blots were performed with antibodies against Flag, HA and Actinin.

The human CST complex exhibits a subunit stoichiometry of 1:1:1 in vivo, with STN1 functioning as a bridge between CTC1 and TEN1 through physical interactions. These direct interactions appear to be crucial for the integrity of the CST complex. Cryo‐electron microscopy (Cryo‐EM) analyses reveal that a single STN1 molecule possesses two distinct interaction sites on CTC1: the N‐terminal OB‐fold domain of STN1 tightly binds to the OB‐G domain of CTC1, while the C‐terminal winged helix‐turn‐helix (wHTH) domain associates with the OB‐E domain of CTC1 with a weaker affinity (Figure 2a) (Lim et al. 2020).

To determine whether the physical interactions with STN1 influence CTC1 stability, we constructed two half‐truncations of STN1 and expressed them in Flag‐CTC1 cells (Figure 2a and S2a). Protein degradation analysis demonstrated that the HA‐tagged STN1_OB_ substantially inhibited the degradation of Flag‐CTC1, extending its half‐life from 3.5 to 8 h, whereas STN1_WT_ extended it to 10 h. In contrast, HA‐STN1_wHTH_ only modestly prolonged the half‐life of Flag‐CTC1 to approximately 5 h, indicating that stronger interactions exert a more pronounced effect (Figures 2b,c and 2b). Additionally, we evaluated the impact of STN1 on a non‐interacting CTC1 mutant. The C‐terminal of CTC1 (OB‐E through OB‐G) serves as a hub for STN1 and TEN1 assembly. A mutation deletion of seven amino acids from OB‐G (1196Δ7) was previously reported to disrupt STN1 interaction (Figure 2a) (Chen et al. 2013). We cloned this mutation and conducted ubiquitylation analysis, which revealed that Flag‐CTC1_1196Δ7_ was also modified by K48‐linked ubiquitin and enriched on ubiquitin‐NTA similar to Flag‐CTC1_WT_ (Figure 2d). However, unlike Flag‐CTC1_WT_, the ubiquitylation of Flag‐CTC1_1196Δ7_ was unaffected by HA‐STN1 expression (Figure 2d). Similarly, HA‐STN1 overexpression did not elevate the protein levels of Flag‐CTC1_1196Δ7_ in HEK293T cells as it did for Flag‐CTC1_WT_ (Figure 2e,f). These results indicate that the physical interaction is essential for STN1 to inhibit CTC1 degradation.

### 3.3 TRIM32 Is Identified as an E3 Ligase for CTC1 Ubiquitylation

**FIGURE 3:**
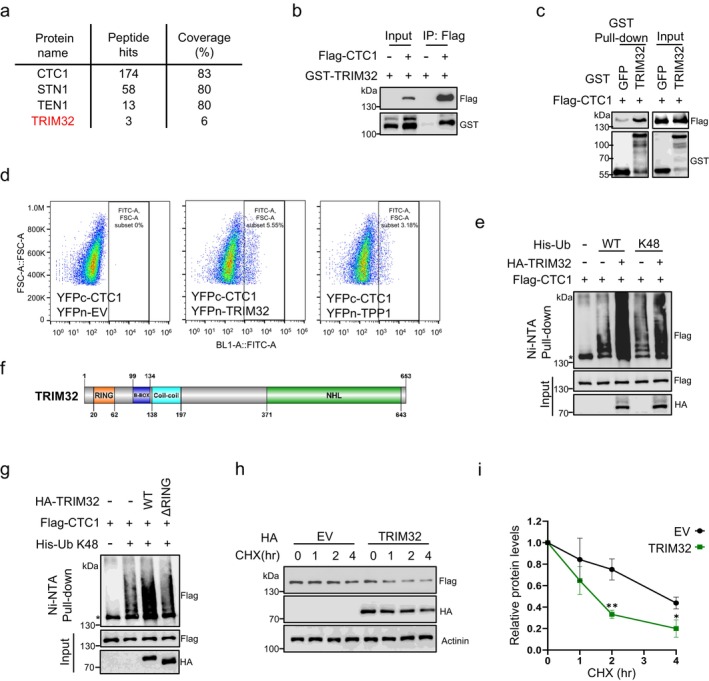
TRIM32 is identified as an E3 ligase for CTC1 ubiquitylation. (a) Mass spectrometry (MS) results of Flag‐immunoprecipitation (IP) from HCT116 cells expressing Flag‐CTC1. (b) IP of Flag‐CTC1 with GST‐TRIM32. Flag‐CTC1 was precipitated from HEK293T cell lysates using Flag M2 beads. (c) In vitro pull‐downs of CTC1 and TRIM32. *E. coli* cells expressed GST‐TRIM32 was added to the Flag‐CTC1‐expressing Sf9 cell lysates, followed by pull‐down using Glutathione‐sepharose beads. (d) Flow cytometry results of the YFP positive signals from BiFC assays. Cells expressing YFPc‐CTC1 plus empty YFPn vectors (YFPn‐EV) or YFPc‐CTC1 plus YFPn‐TPP1 were used as negative or positive controls, respectively. (e) In vivo ubiquitination assays showing TRIM32's effects on the WT or K48‐linked ubiquitin level of Flag‐CTC1. Immunoblot of Ni‐NTA pull‐downs and whole‐cell lysates input from HEK293T cells co‐transfected with Flag‐CTC1, His‐Ub (WT or K48) and HA‐TRIM32. (f) Cartoon showing the structure of TRIM32. (g) In vivo ubiquitination assays showing the effects of WT or ΔRING‐mutated TRIM32 on K48‐linked Flag‐CTC1 ubiquitination. Immunoblot of Ni‐NTA pull‐downs and whole‐cell inputs from HEK293T cells co‐transfected with Flag‐CTC1, K48 His‐Ub and constructs encoding HA‐tagged WT or ΔRING TRIM32. (h) Protein degradation analysis of Flag‐CTC1 in HEK293T cells transfected with or without HA‐TRIM32 over a 4‐h period. (i) Quantification of relative Flag‐CTC1 levels from (h). *N* = 3 independent experiments. Error bars represent mean ± S.E.M. Western blots were performed with antibodies against Flag, HA and Actinin. *: Non‐specific binding to Ni‐NTA resin.

While STN1 significantly regulates CTC1 ubiquitylation, it lacks direct catalytic activity. To identify the catalytic enzyme, we performed immunoprecipitation of Flag‐CTC1 followed by mass spectrometry analysis of the IP products. The results revealed the identification of peptides corresponding to multiple E3 ubiquitin ligases, in addition to STN1, TEN1, and the subunits of DNA polymerase α‐primase (pol α) (Figures 3a and S3a). Notably, among the top four E3 ligases, TRIM32 demonstrated the capacity to augment the ubiquitination levels of CTC1 (Figure S3b). The association between TRIM32 and CTC1 was confirmed through in vivo co‐immunoprecipitation and in vitro pulldown assays. In vivo, GST‐tagged TRIM32 co‐precipitated with Flag‐CTC1 from HEK293T cells (Figure 3b). In vitro, SF9 cells expressed Flag‐tagged CTC1 was effectively co‐pulled down by GST‐TRIM32 purified from *E. coli* cells using glutathione agarose (Figures 3c and S3c). Additionally, we observed TRIM32‐CTC1 interactions in situ using bi‐molecular fluorescence complementation (BiFC) assays. In these assays, interactions between two proteins that are tagged with YFP fragments (YFPn or YFPc) will bring the two halves of YFP to close proximity for fluorescence complementation and detection (Figure S3d) (Morell et al. 2008). As a result, co‐expression of TRIM32 and CTC1 tagged with YFP fragments in HEK293T cells yielded YFP‐positive signals, comparable to those seen in cells co‐expressing CTC1 and TPP1 (Figures 3d and S3e). These findings validate the existence of the TRIM32‐CTC1 association.

We subsequently examined the effects of TRIM32 on CTC1 ubiquitination and degradation. In vivo ubiquitylation analysis showed that HA‐tagged TRIM32 enhanced the enrichment of Flag‐CTC1 on ubiquitin NTA when co‐expressed with either WT or K48 ubiquitin (Figure 3e). We also performed in vitro ubiquitylation assays to verify TRIM32's capability to modify CTC1 using purified His‐tagged TRIM32 (Figure S3f) in conjunction with E1 and E2 ubiquitin‐activating enzymes, along with K48‐linked ubiquitin and ATP. Due to CTC1's instability, we could only purify CTC1 from SF9 cells when co‐expressed with STN1 (Figure S3g). Western blot analysis revealed that His‐TRIM32 effectively modified Flag‐CTC1 with K48‐linked polyubiquitin chains in vitro, despite the presence of STN1 (Figure S3h). To further confirm TRIM32 as an E3 ligase for CTC1, we constructed a catalytically inactive mutant lacking the RING‐finger domain (ΔRING) based on the reported structure of TRIM32 (Figure 3f) and assessed its influence on CTC1 ubiquitylation. In ubiquitylation assays, unlike HA‐TRIM32_WT_, HA‐TRIM32_ΔRING_ did not enhance K48‐linked ubiquitylation of CTC1, indicating that the catalytic domain of TRIM32 is essential for CTC1 ubiquitylation (Figure 3g). Consistently, TRIM32 overexpression expedited Flag‐CTC1 degradation, reducing its half‐life to approximately 1.5 h (Figure 3h,i).

### 3.4 Lys776 Is Essential for CTC1 Ubiquitination

**FIGURE 4:**
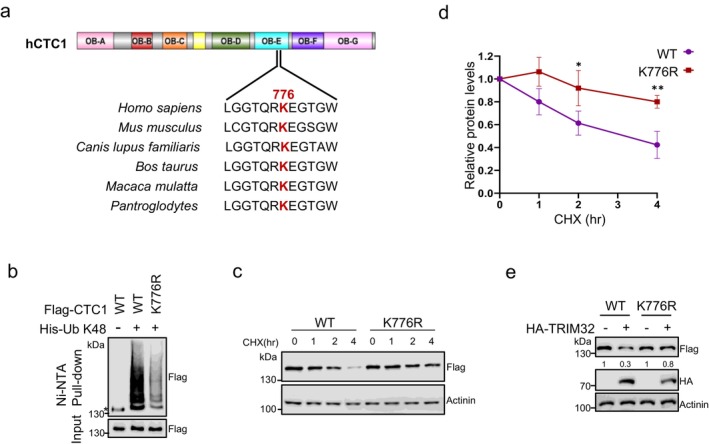
Lys776 is essential for CTC1 ubiquitination. (a) Sequence alignment showing that K776 is conserved in higher eukaryotes. K776 is highlighted in red. (b) In vivo ubiquitination assays showing the K48‐linked ubiquitin level of WT and K776R mutated Flag‐CTC1. Immunoblot of Ni‐NTA pull‐downs and whole‐cell inputs from HEK293T cells co‐transfected with WT or K776R Flag‐CTC1 and K48 His‐Ub. *: Non‐specific binding to Ni‐NTA resin. (c) Protein degradation analysis of WT and K776R Flag‐CTC1 in HEK293T cells treated with CHX over a 4‐h period. (d) Quantification of relative Flag‐CTC1 levels from (c). *N* = 3 independent experiments. Error bars represent mean ± S.E.M. (e) Immunoblot WT and K776R Flag‐CTC1 levels in HEK293T cells transfected with or without HA‐TRIM32. Numbers show the quantification of the relative amount of Flag‐CTC1. Western blots were performed with antibodies against Flag, HA and Actinin.

To elucidate the mechanism underlying CTC1 ubiquitylation, we employed mass spectrometry to identify potential ubiquitination sites. Flag‐CTC1 stably expressing HEK293T cells were co‐transfected with K48‐linked ubiquitin, with or without TRIM32. The analysis identified Lys‐776 (K776) as a proximal ubiquitination residue, with the percentage of modified peptide hits increasing 1.3 times in samples expressing TRIM32 compared to control (Figure S4a,b). K776 is located within the OB‐E domain of CTC1. Sequence alignment of CTC1 across various vertebrate species revealed that this residue is highly conserved (Figure 4a). Mutation of K776 to arginine (R) significantly reduced the K48‐linked ubiquitylation levels of Flag‐CTC1 in ubiquitylation assays (Figure 4b). We then generated a Flag‐tagged CTC1_K776R_ stable expressing HEK293T cell line to assess the stability of the mutant. Analysis of protein half‐life over a 4‐h period revealed that Flag‐CTC1_K776R_ degraded much more slowly compared to Flag‐CTC1_WT_ (Figure 4c,d). Additionally, TRIM32 overexpression had a diminished effect on the degradation rate and ubiquitylation levels of Flag‐CTC1_K776R_ (Figures 4e and S4c). These results suggest that K776 is critical for CTC1 ubiquitylation and degradation. Notably, the levels of Flag‐CTC1_K776R_ still gradually decreased during half‐life analysis (Figure 4c,d). Furthermore, TRIM32 slightly accelerated Flag‐CTC1_K776R_ degradation (Figure 4e), indicating that K776 may not be the only ubiquitination site on CTC1, or that alternative degradation pathways exist.

### 3.5 STN1 Inhibit CTC1 Ubiquitination and Degradation by Competing With TRIM32 for CTC1 Binding

**FIGURE 5:**
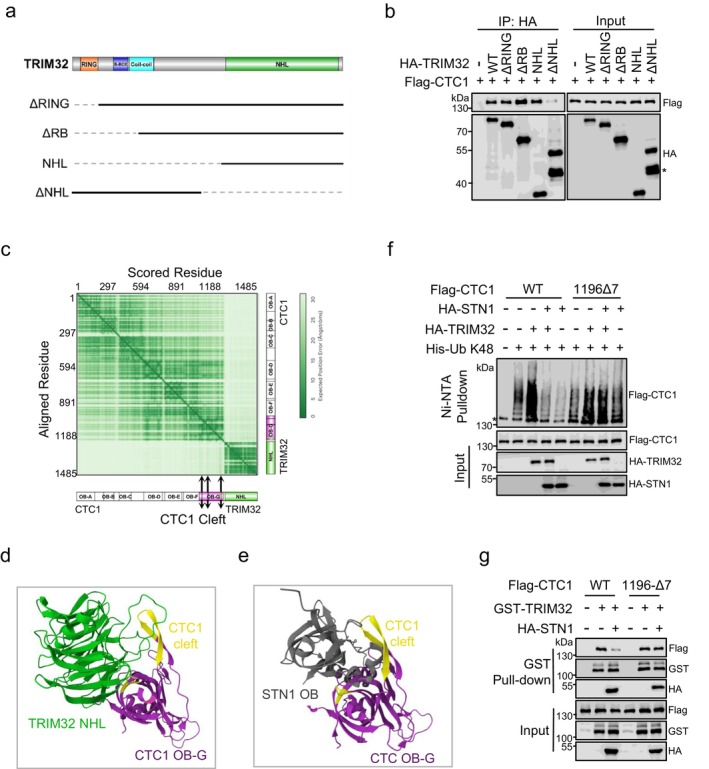
STN1 competes with TRIM32 for CTC1 binding to inhibit CTC1 ubiquitination and degradation. (a) Schematic representation of the design of TRIM32 mutants. (b) IP of Flag‐CTC1 with HA‐tagged TRIM32 and mutants. HA‐TRIM32 was precipitated from HEK293T cell lysates using HA beads. *: Degradation bands. (c) Predicted aligned error (PAE) plot from AlphaFold3 modeling of CTC1‐TRIM32. Representative of five ranked models generated with default parameters. Arrows indicate CTC1 Cleft motif region. (d‐e) Predicted structures of a TRIM32‐NHL (d) or STN1‐OB (e) bound to CTC1. Only the OB‐G of CTC1 is shown. (f) In vivo ubiquitination assays showing the effects of STN1 on TRIM32's function in K48‐linked ubiquitination of WT and 1196Δ7 Flag‐CTC1. Immunoblot of Ni‐NTA pull‐downs and whole‐cell inputs from HEK293T cells co‐transfected with Flag‐CTC1 WT or 1196Δ7, K48 His‐Ub, HA‐TRIM32 and HA‐STN1. *: Non‐specific binding to Ni‐NTA resin. (g) GST pull‐downs of WT or 1196Δ7 Flag‐CTC1 with GST‐TRIM32 in the present or absent of HA‐STN1 from HEK293T cell lysates using Glutathione‐sepharose beads. Western blots were performed with antibodies against Flag, GST and HA.

The contrasting regulatory roles of STN1 and TRIM32 in K48‐linked ubiquitination and degradation of CTC1 suggest that they may compete for binding sites on CTC1. To investigate how TRIM32 binds to CTC1, we performed AlphaFold3 (AF3) protein interaction predictions. The results revealed an interaction between the C‐terminal NHL domain of TRIM32 and the OB‐G domain of CTC1 (Figure S5a,b). Co‐immunoprecipitation assays confirmed that TRIM32 truncations containing the NHL domain, including the NHL domain alone, could bind to CTC1, whereas the NHL deletion mutant (ΔNHL) barely does (Figure 5a,b).

We then analyzed the interaction of TRIM32‐NHL with CTC1. Interestingly, TRIM32‐NHL was predicted to interact with high confidence with the “cleft” motif on CTC1 OB‐G, a region previously identified as a critical binding site for STN1‐OB (Figures 5c and S5c) (Lim et al. 2020). We also visualized the interaction between STN1‐OB and CTC1 OB‐G, with the model aligning well with the CST cryo‐EM structure, further validating the predictions (Figure S5d,e) (Lim et al. 2020). Next, we compared the interactions between TRIM32‐NHL and OB‐G with the binding model of STN1‐OB and OB‐G (Figure S5c,d). Close‐up views indicated that TRIM32‐NHL and STN1‐OB engage the same surface on CTC1‐OB‐G (Figure 5d,e).

To examine whether STN1 inhibits TRIM32's ability to promote CTC1 ubiquitylation, we performed ubiquitylation assays and found that HA‐TRIM32 significantly increased the enrichment of Flag‐CTC1_WT_ on K48‐linked Ub‐NTA, but this enhancement was abolished in the presence of HA‐STN1 (Figure 5f). Notably, HA‐STN1 did not influence the increased ubiquitylation of Flag‐CTC1_1196Δ7_ stimulated by TRIM32, indicating that STN1's physical interaction protects CTC1 from TRIM32‐mediated ubiquitylation. Consistently, in vivo GST pulldown assays demonstrated that HA‐STN1 expression reduced the amount of Flag‐CTC1_WT_ interacting with GST‐TRIM32, while having minimal effect on the interaction between Flag‐CTC1_1196Δ7_ and GST‐TRIM32 (Figures 5g and S5f). Together, these data support our hypothesis that STN1 competes with TRIM32 for binding sites on CTC1, thereby preventing CTC1 ubiquitylation and degradation.

### 3.6 TRIM32 Promotes Somatic Cell Early Senescence

**FIGURE 6:**
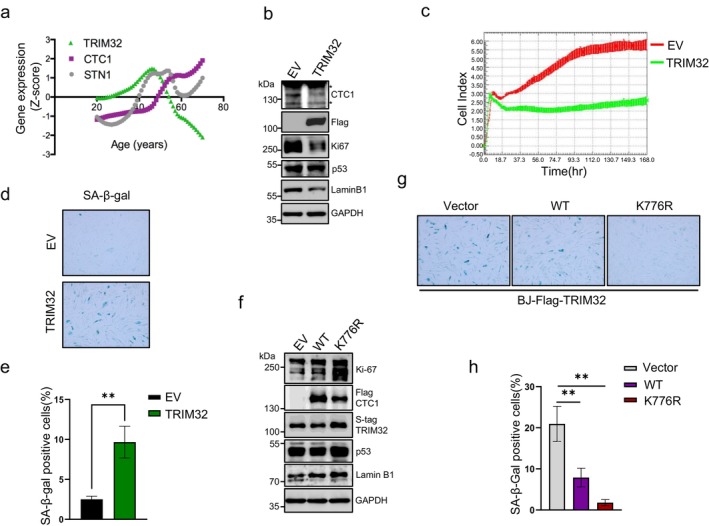
TRIM32 promotes somatic cell aging and early senescence. (a) Graph showing the relative levels of CTC1/STN1/TRIM32 mRNA expression in BJ fibroblast cells from individuals of the indicated ages. (b) Immunoblot showing the protein levels of CTC1, p53, Ki67 and LaminB1 in BJ cells over‐expressing Flag‐tagged TRIM32. *: Cross‐reacting bands. (c) Representative Cell Index profile from real‐time proliferation analysis of BJ cells stably expressing Flag‐TRIM32 at PD33 using xCELLigence RTCA. Cells were monitored for the indicated time (*n* = 3). (d, e) Early senescence induced by TRIM32 in BJ cells. BJ cells were infected with lentivirus containing empty vectors (EV) or SFB‐TRIM32‐expressing vectors and selected for puromycin resistance at PD17. Senescence‐associated β‐galactosidase (SA‐β‐gal) staining was performed at PD30. (d) Representative images of the SA‐β‐gal staining. (e) Quantification of senescence cells (with blue stain) from (d). (f) Immunoblot showing the protein levels of p53, Ki‐67 and Lamin B1 in TRIM32 stable expressing BJ cells with wild‐type CTC1 or K776R mutant expression. *: Cross‐reacting bands. (g) SA‐β‐gal staining of BJ cells from (f). (h) Quantification of senescence cells (with blue stain) from (g).

Because of the important roles of CST in cell proliferation, the depletion of either CTC1 or STN1 induced cell senescence (Feng et al. 2017; Huang et al. 2012). To further investigate the relationship between CST expression and human aging, we analyzed the change of CST and TRIM32 levels during somatic cells aging. The gene expression data of BJ fibroblasts derived from healthy individuals across different ages were extracted from the large RNA‐seq dataset of Genotype‐Tissue Expression (GTEx) project using voyAGEr database (The Genotype‐Tissue Expression (GTEx) project 2013). As depicted in Figures 6a and S6a, the expression of CTC1, STN1 and TEN1 gradually increased after the age of 60, coinciding with the onset of Lamin B1 decline, indicating that greater CST levels are required in older cells. Conversely, TRIM32 expression was found to decline starting around the same age, suggesting a contrasting regulatory role for TRIM32 in cell aging.

Given its function in stimulating CTC1 degradation, it is not surprising that TRIM32 negatively impacts cell proliferation. To inspect these effects, we overexpressed exogenous SFB‐tagged TRIM32 in BJ fibroblasts. As anticipated, a dramatic reduction in CTC1 protein levels was observed (Figure 6b). The levels of cell proliferation markers, such as Ki67 and LaminB1, also decreased in TRIM32 overexpression cells (Figure 6b). Increasing DNA damage signals on telomeres were also observed in these cells (Figure S6b,c). We then monitored the cell proliferation with a Real‐Time Cell Analysis (RTCA) system. The results showed that TRIM32 overexpressing significantly delimited the growth of BJ cells, especially for the later passages (PD33 and PD36 comparing to PD20) (Figures 6c and S6d,e). Furthermore, the senescence‐associated β‐galactosidase (SA‐β‐Gal) staining demonstrated a three‐time increase in the number of senescent cells following TRIM32 overexpression at PD30 (Figure 6d,e), indicating that TRIM32 acts as a positive regulator of cellular senescence in somatic cells.

Because TRIM32 functions as an E3 ubiquitin ligase with broad tissue expression and numerous substrates (Sun et al. 2025; Thakur et al. 2025; Zhan et al. 2025), we sought to determine whether its effect on cellular senescence is mediated specifically through the modification of CTC1. To this end, we overexpressed either exogenous wild‐type CTC1 or the ubiquitination‐resistant mutant (K776R) in the TRIM32‐overexpressing cells and assessed their ability to rescue the senescence‐associated phenotypes. As a result, expression of the K776R mutant markedly suppressed the reduction of proliferation markers Ki67 and Lamin B1, whereas wild‐type CTC1 exerted a comparatively weaker effect (Figure 6f). Consistently, SA‐β‐Gal staining was more substantially reduced in cells expressing the CTC1 K776R mutant compared to those expressing wild‐type CTC1 (Figure 6g,h). Thus, all evidence aligns with our hypothesis that TRIM32 promotes early senescence by stimulating CTC1 ubiquitin‐mediated degradation in somatic cells.

## 4 Discussion

In this study, we identified TRIM32 as the first ubiquitin E3 ligase associated with the human CST complex. Overexpression of TRIM32 promotes the proteasomal degradation of CTC1 and induces early senescence in somatic cells. In contrast, STN1 prevents the ubiquitination and degradation of CTC1, thereby stabilizing the complex. Using AlphaFold3 for interaction prediction, we hypothesized that TRIM32 binds to the same “cleft” motif on the OB‐G domain of CTC1 as STN1, which provides an explanation for how STN1 competes with TRIM32 and inhibits its ability to modify and degrade CTC1.

**FIGURE 7:**
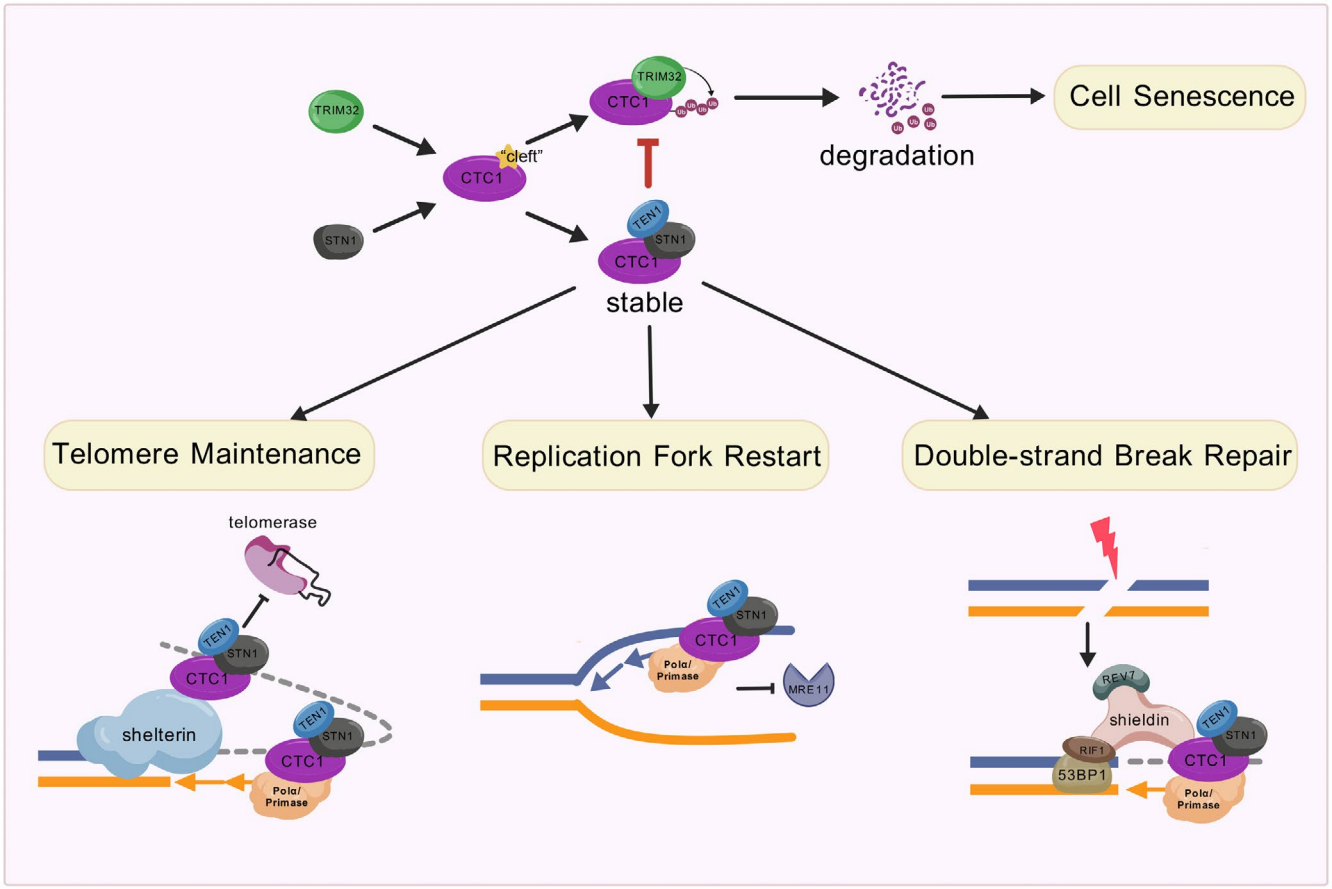
Working model depicting how STN1 safeguards CTC1 from TRIM32‐mediated ubiquitin‐proteasome degradation, thereby preserving CST complex integrity and its roles in telomere maintenance, DNA replication, and DNA damage repair.

The CST complex, initially identified as an accessory factor of DNA polymerase α‐primase (pol α) (Casteel et al. 2009), has been shown to play multiple essential roles in cell proliferation (Figure 7) (Feng et al. 2017). At telomeres, CST binds to the single‐stranded DNA (ssDNA) overhangs, assists with telomerase termination (Chen et al. 2012; Feng et al. 2018; H. Wang et al. 2023), and facilitates pol α in the synthesis of C‐strands (Feng et al. 2017; Huang et al. 2012; Zaug et al. 2022). During genome replication, CST localizes to stalled replication forks, protects nascent‐strand DNA from degradation (Jaiswal et al. 2023; Lyu et al. 2021), aids in resolving difficult‐to‐replicate DNA structures (such as G‐quadruplexes) (Bhattacharjee et al. 2017; Li et al. 2023; Zhang et al. 2019), and recruits pol α to restart replication (Y. Wang et al. 2019). At sites of double‐strand breaks, CST counteracts DNA resection by promoting pol α‐mediated filling of the DNA gaps (King et al. 2025; Mirman et al. 2022). Depletion of either CTC1 or STN1 impairs these functions, leading to cellular senescence (Feng et al. 2017; Huang et al. 2012). Therefore, aging cells—characterized by shorter telomeres and increased DNA damage—may become more reliant on CST to maintain genome stability. Consistently, our analysis of large RNA‐seq datasets from healthy individuals in the GTEx database revealed that the expression of CTC1 and STN1 in somatic cells (BJ fibroblasts) from individuals over 60 years old gradually increases with age.

Conversely, TRIM32 functions as a negative regulator of cell proliferation in BJ fibroblasts. Its overexpression leads to a marked reduction in CTC1 levels, which in turn induces senescence in BJ fibroblasts (Figure 7). Although TRIM32 has been reported to mediate the degradation of p53 (Liu, Zhang, et al. 2014), a critical regulator of cell cycle progression and apoptosis, we did not observe any significant alterations in p53 levels in our Flag‐TRIM32‐overexpressing BJ fibroblast cells (Figure 6b). Furthermore, the role of TRIM32 in cancer cells has been shown to suppress p53 expression, thereby inhibiting senescence (Liu, Zhang, et al. 2014). However, in somatic cells, our data demonstrate that TRIM32 overexpression promotes cell senescence. These findings suggest that TRIM32 may exert divergent effects on senescence in somatic cells compared to cancer cells.

## Author Contributions

Y.L. designed the project and performed most of the experiments. X.L. performed the senescence‐related assays with BJ cells. G.K. performed AlphaFold analysis and purified the CST complex. T.M. assisted with tissue culture, western blotting, and protein purification. F.Z. performed the Flag‐CTC1 IP‐MS. Z.H. assisted with AlphaFold data analysis. H.W. initiated this work and guided ubiquitylation assays. Z.L. provided information, reagents, and advice. X.F. conceived the study and wrote the paper with input from all co‐authors.

## Conflicts of Interest

The authors declare no conflicts of interest.

## Supporting information


**Figure S1:** acel70214‐sup‐0001‐FigureS1.docx.

## Data Availability

Data sharing not applicable to this article as no datasets were generated or analyzed during the current study.
