# Supplementary material for: STN1 Shields CTC1 From TRIM32‐Mediated Ubiquitination to Prevent Cellular Aging
Source: Aging Cell. 2025 Sep 9;24(11):e70214. doi: 10.1111/acel.70214 (PMC12608094; doi:10.1111/acel.70214)
Supplement: Supplementary file 1 — Figure S1: acel70214‐sup‐0001‐FigureS1.docx. [file ACEL-24-e70214-s001.zip › acel70214-sup-0001-FigureS1-S7@Supplemental Figure legends.docx]

**Supplemental Figures**

**Figure S1. STN1 has minimal effect on CTC1 mRNA levels.** (a-b) Relative mRNA levels of CTC1 following STN1 knockdown (a) or overexpression (b) in HCT116 cells. (c) Two repeats of Fig. 1d showing Flag-CTC1 and control protein levels over a 12-hour period with or without MG132 in the presence of CHX to inhibit protein synthesis.

**Figure S2. Effects of STN1 truncations on CTC1 protein levels.** (a) WB showing the expression of HA-tagged STN1 constructs in HEK293T cells. (b) Two repeats of Fig. 2b showing protein degradation analysis of Flag-CTC1 in HEK293T cells transfected with the indicated HA-STN1 mutants over a 12-hour period. *: degradation bands.

**Figure S3. TRIM32 is identified as the E3 ligase for CTC1 ubiquitination.** (a) A list of Top 10 and essential enrichments from CTC1-IP MS assay. (b) *In vivo* ubiquitination assays showing the effects of indicated E3 ligases on CTC1 ubiquitination levels. (c) Coomassie blue-stained SDS-PAGE gel displaying the protein levels of GST-TRIM32 purified from *E. coli* cells. (d) Schematic representation of the strategy of BiFC assays. (e) WB showing the expression of YFP-tagged protein levels used in BiFC assays. *: cross-reacting bands. (f) Silver-stained SDS-PAGE gel showing protein levels of His-TRIM32 purified from *E. coli* cells. (g) Silver-stained SDS-PAGE gel displaying the protein levels of Flag-CTC1 and His-STN1 purified from Sf9 cells. (h) WB of *in vitro* ubiquitination assays using purified CTC1-STN1, commercial E1, E2 and Ub-K48 proteins, with or without purified His-TRIM32 from *E. coli* cells.

**Figure S4. TRIM32 promotes K48-linked ubiquitination of CTC1 on K776.** (a) Representative plots of relative fragment intensity versus m/z for fragmentation data from MS identifying K48-Ub on CTC1 in the presence of TRIM32. The modified lysine residue is highlighted in red. (b) Quantification of CTC1 peptide hits intensity, comparing modified and unmodified K776 sites from MS sequencing with or without TRIM32. (c) *In vivo* ubiquitination assays showing the K48-linked ubiquitin level of WT and mutant Flag-CTC1. Immunoblots of Ni-NTA pull-downs and whole-cell inputs from HEK293T cells co-transfected with WT or mutated Flag-CTC1, K48 His-Ub and HA-TRIM32. Western blots were performed with antibodies against Flag, HA and Actinin.

**Figure S5. AlphaFold3 interaction prediction of TRIM32’s binding to CTC1.** (a) Predicted structures of a complex comprising full-length CTC1 (purple) and TRIM32 (grey). The predicted interaction domains of them are highlighted in dark purple and dark green, respectively. (b) pLDDT plot showing per-residue confidence scores across TRIM32 from the ranked TRIM32-CTC1 models. (c-e) Predicted structures of TRIM32-NHL (c) or STN1-OB (d) bound to CTC1, and the cryo-EM structure of the CTC1-STN1-TEN1 complex (e). (f) Two repeats of Fig. 5g showing

GST pull-downs of WT or 1196Δ7 Flag-CTC1 with GST-TRIM32 in the present or absent of HA-STN1 from HEK293T cells.

**Figure S6. TRIM32 overexpression inhibits cell proliferation in BJ fibroblasts.** (a) Graph showing the relative levels of p53/LaminB1/CTC1/STN1/TEN1/TRIM32/GAPDH mRNA expression in BJ fibroblast cells from individuals of the indicated ages. (b) Representative images of the telomere-dysfunction-induced foci (TIF) analysis in empty vector (EV) control and TRIM32 overexpression BJ cells using anti-γH2AX (red) and anti-TPP1 (green) antibodies. Arrows indicated superimposable foci. (c) Quantification of cells with γH2AX and TPP1 co-staining foci (TIFs) from (b). (d-e) Representative Cell Index profiles from real-time proliferation analysis of BJ cells stably expressing Flag-TRIM32 at PD20 (d) and PD36 (e) using xCELLigence RTCA. Cells were monitored for the indicated time.

**Figure S7. Raw data of the indicated immunoblots from the main figures.**
